# Ludwig's angina and acute myocardial infarction: A case report

**DOI:** 10.1002/ccr3.7832

**Published:** 2023-08-24

**Authors:** Ronald Kato, Umar Ssebagala, Kainembabazi katrina

**Affiliations:** ^1^ Department of Emergency Medicine Savannah Hospital Nairobi Kenya; ^2^ Department Of Internal Medicine King David Hospital Nairobi Kenya; ^3^ Department of Health sciences North Hennepin Community College Brooklyn Park Minnesota USA

**Keywords:** cardiac markers, ischemic pain, lymph nodes, myocardial infarction, polymicrobial

## Abstract

Ludwig's angina was first described in 1839 by German physician, Wilhelm Frederick Von Ludwig as a rapidly and fatal progressive gangrenous cellulitis and edema of the soft tissues of the neck and floor of the mouth with rapid spread to other places like anterior mediastinum. However, Type 2 acute myocardial infarction (MI) due to Ludwig's angina has not been documented. A 62‐year‐old male presented to the emergency department with visible anterior neck swelling for 1 week, which was preceded by a tooth arch 1 week prior, the patient presented with a high grade fevers, dysphonia, dysphagia, and facial swelling. No history of trauma. He reported in the past 24 h prior to evaluation, a steady progression of pain intensity with rapid progression and anterior neck skin erythema and swelling. The pain was exacerbated by rotation of the neck, tongue protrusion, and speaking. On examination, there was a visible anterior neck swelling measuring 10.0 × 3.0 cm in widest dimensions, exquisitely tender to palpation with a positive temperature gradient, skin hyperpigmentation and firm in consistency, no crepitus, fluctuance, or induration. Tongue appeared elevated with sublingual edema and pooling of secretions. No stridor. A chest and neck ultrasound scan revealed an extensive abscisic mass from the submandibular, neck, sternal notch, and right clavicular region with the largest pockets measuring 2.11 × 0.8 cm, 2.03 × 0.62 cm, 1.50 × 1.1 cm, with noted submandibular, subclavicular and deep and superficial cervical lymph nodes, the largest measuring 1.23 × 1.63 cm in dimensions. A neck‐CT scan with contrast revealed a pronounced subcutaneous tissue localized collection extending to both submandibular spaces measuring about 5.5 × 12.5 × 9.5 cm with mural enhancement. The upper chest cuts showed moderate pleural effusions and a paracardial hypodense well‐defined lesion measuring 7.5 × 2.5 cm with mild pericardial effusion. The patient was referred to the ear, neck and throat, ENT surgeon for urgent drainage of the abscess, which was done successfully and about 300 mL of hemorrhagic pus was drained. Then transferred to highly dependent unit, (HDU) for IV antibiotic administration and vital observations, prior to that electrocardiogram, ECG showed a normal sinus rhythm. The following day in HDU, the patient started experiencing a chest pain of sudden onset radiating to the upper jaws, left forearm and throbbing in nature, palpitations and started becoming diaphoretic. Blood pressure was 150/70 mmgh and pulse of 120 bpm. ECG readings demonstrated ST‐elevation, at lead 11, V2, and V3, cardiac Troponin I and CK‐MB were elevated 10.0 ng/mL, (<0.4 ng/mL) and 150.0 IU/L, (5‐25 IU/L) respectively. The patient was started on medications to relief acute ischemic pain these included, sublingual nitroglycerin 0.6 mg, morphine 5 mg intravenous slowly, antithrombotic, and beta‐adrenergic blockade. He was kept in HDU later with heparin 80 U/kg bolus and 8 U/kg continuous infusion and was taken for coronary angiogram which demonstrated no any coronary artery occlusion. The patient later on started to register improvement and later discharged on medications for follow‐up. On follow‐up, the subsequent ECG showed persistent atrial fibrillation and patient was discharged on P2Y12 inhibitor, clopidogrel, 75 mg, and beta‐blocker, metoprolol 50 mg. Over the past three decades, mortality rates for acute MI have increased significantly, One common subtype, type 2 MI is noted and driven by a myocardial oxygen supply and demand mismatch in the absence of coronary thrombosis. T2MI can occur with or without obstructive coronary disease like in this patients with angiographically normal coronary arteries. T2MI is increasingly recognized because of various septic pathophyisologies that cause increased myocardia oxygen demand. Evidence of myocardial ischemia especially those with sepsis are likely to develop myocardial injury. T2MI is frequent and explains a significant increase in clinical practice. A consensus is needed about how the diagnosis is established, to facilitate evidence‐based therapies geared toward improving outcomes.

## Graphical abstract

Visible anterior neck swelling. Ultrasound scan shows extensive submandibular abscisic mass.

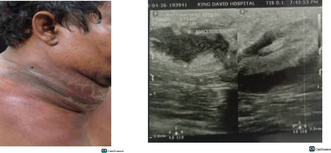

## 1 INTRODUCTION

Ludwig's Angina was first described in 1839 by German Physician, Wilhelm Frederick Von Ludwig as a rapidly and frequently fatal progressive gangrenous cellulitis and edema of the soft tissues of the neck and floor of the mouth. Ludwig's is often loosely applied to deep space neck infections but should be limited to those infections which are bilateral and involves the submandibular spaces including both sublingual and sub‐mylohyoid spaces, and has rapid spread to other places like anterior mediastinum. However, Type 2 myocardial infarction (MI) due to Ludwig's angina has not been documented.^1^

## 2 CASE REPORT

A 62‐year‐old male a referral from a peripheral facility presented to the emergency department with visible anterior neck swelling for 1 week, which was preceded by a tooth arch 1 week prior, the patient presented with a high grade fevers, dysphonia, dysphagia, and facial swelling. No any history of facial or neck trauma. The patient however reported a poor dental hygiene. No any history of chronic illnesses like diabetes, hypertension.

The patient reported in the past 24 h prior to evaluation, he noted steady progression of pain intensity with rapid progression in the 24 h with anterior neck skin erythema and swelling. He stated that the pain was exacerbated by rotation of the neck, tongue protrusion, and speaking.

**FIGURE 1:**
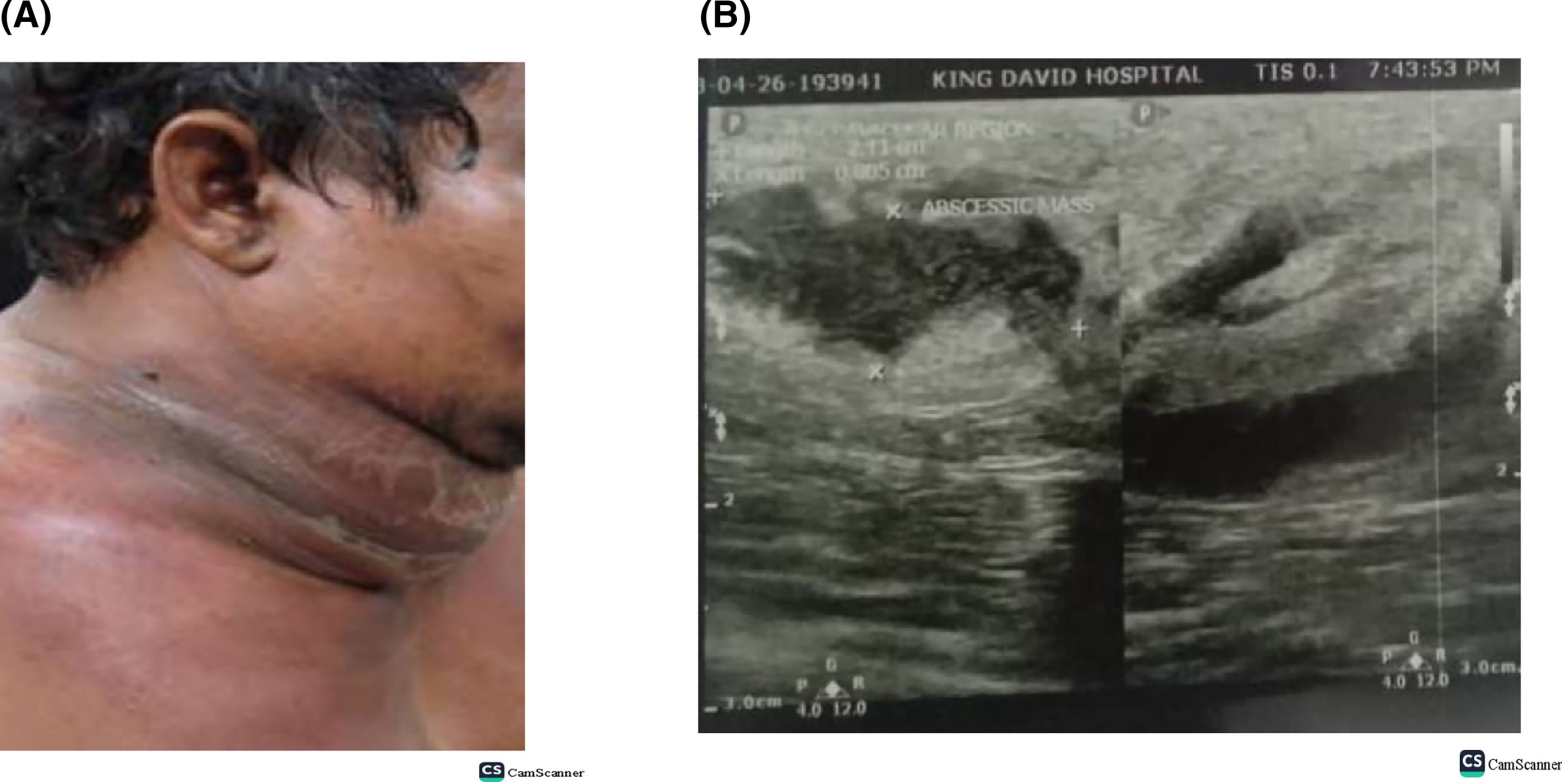
(A) visible anterior neck swelling. (B) Ultrasound scan shows extensive submandibular abscisic mass. 10.0 cm × 3.0 cm in widest dimensions. Largest pockets measuring 2.11 × 0.8 cm. Skin hyperpigmentation.

On examination, there was a visible anterior neck swelling measuring 10.0 × 3.0 cm in widest dimensions, exquisitely tender to palpation with a positive temperature gradient, skin hyperpigmentation and firm in consistency, no crepitus, fluctuance, or induration (Figure 1A). Tongue appeared elevated with sublingual edema and pooling of secretions. No stridor. Other systemic examination was essentially unremarkable.

A chest and neck ultrasound scan revealed an extensive abscisic mass from the submandibular, neck, sternal notch and right clavicular region with the largest pockets measuring 2.11 × 0.8 cm, 2.03 × 0.62 cm, 1.50 × 1.1 cm, with noted submandibular, subclavicular and deep and superficial cervical lymph nodes noted, with the largest measuring 1.23 × 1.63 cm in dimensions (Figure 1B).

**FIGURE 2:**
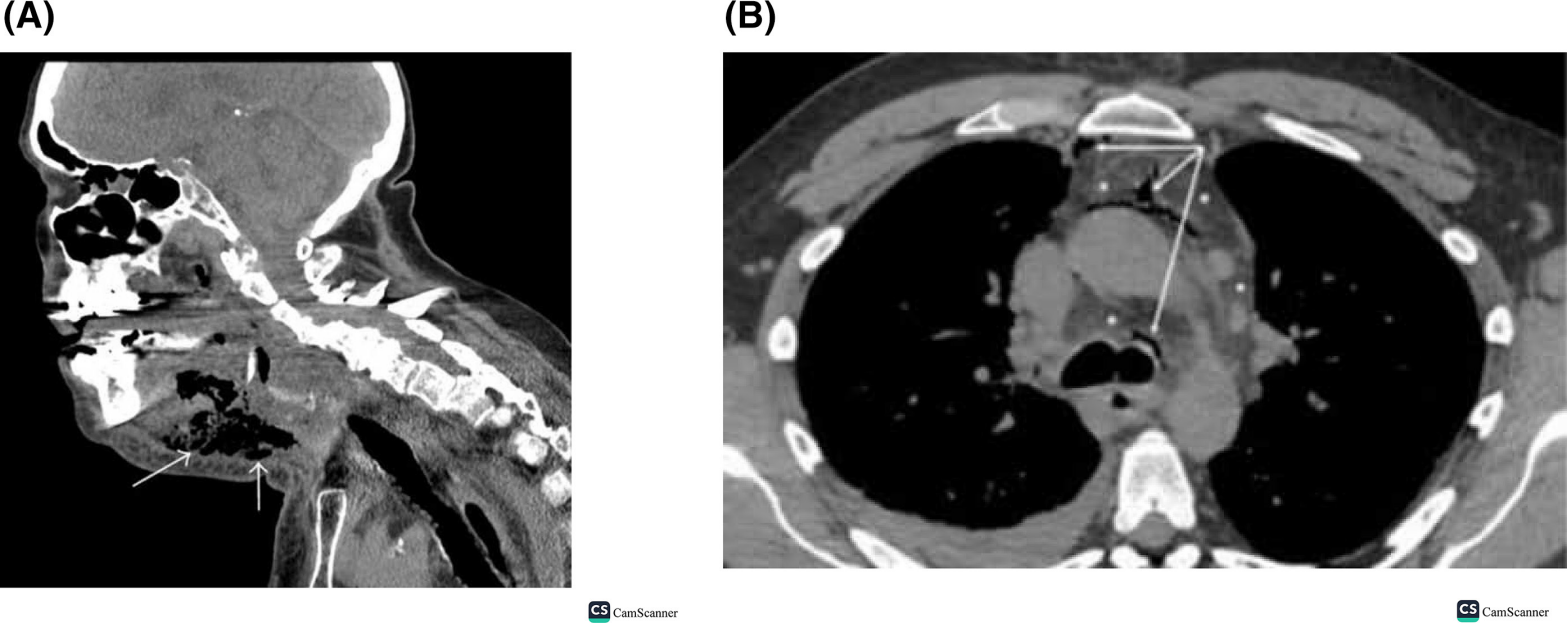
(A) A neck‐CT scan with Contrast. (B) The upper chest with mild pleural effusions. Revealed a pronounced subcutaneous tissue paracardial hypodense lesion and mild pericardial effusion. Midline localized collection detected extending to both submandibular spaces measuring about 5.5 × 12.5 × 9.5 cm with mural enhancement.

**FIGURE 3:**
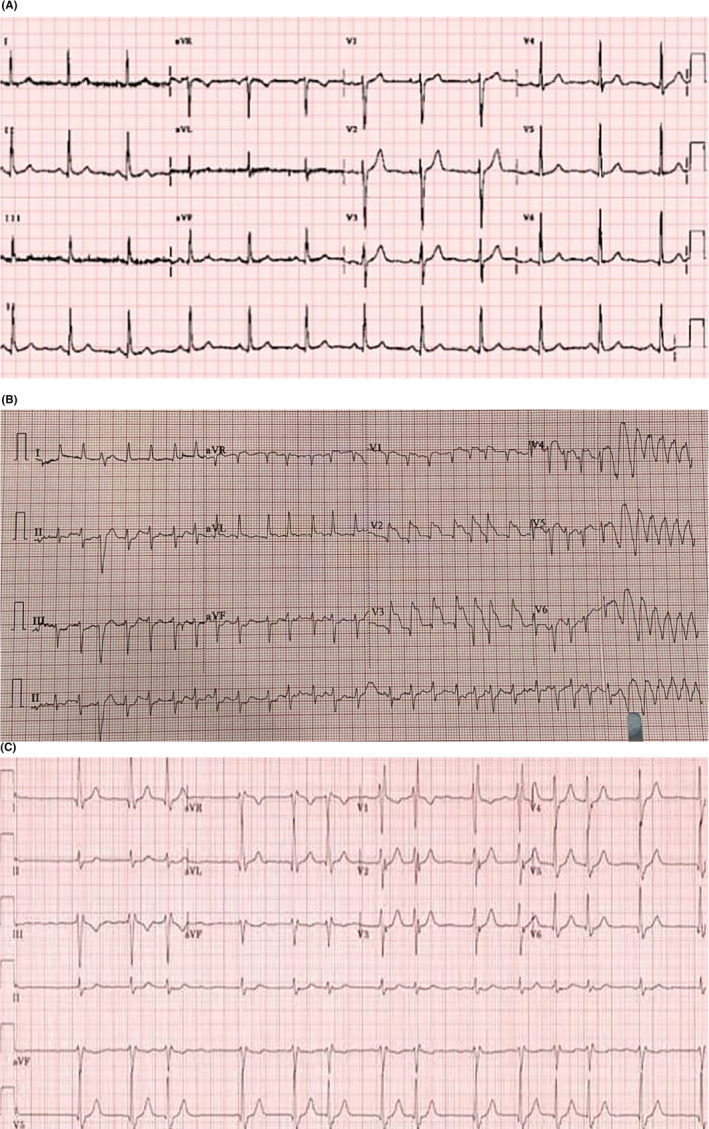
(A) Normal ECG showing sinus rhythm prior to admission. (B) ECG readings demonstrated ST‐elevation, at lead 11, V2, and V3. (C) Showing persistent atrial fibrillation with sinus tachycardia after discharging the patient.

A neck‐CT scan with contrast was requested and it revealed a pronounced subcutaneous tissue midline localized collection detected extending to both submandibular spaces measuring about 5.5 × 12.5 × 9.5 cm with mural enhancement. The upper chest cuts showed moderate pleural effusions and a paracardial hypodense well‐defined lesion measuring 7.5 × 2.5 cm with mild pericardial effusion (Figure 2A,B). Preadmission electrocardiogram, ECG was done and showed normal sinus rhythm (Figure 3A).

The patient was referred to the ENT surgeon for urgent drainage of the abscess, which was done successfully and about 300 mL of hemorrhagic pus was drained of about 500 mL. Fluid resuscitations and culture and sensitivity was done and revealed polymicrobial infection with *streptococcus pyogenes* and *staphylococcus aureus* bacterial species. The patient was transferred to HDU for intravenous, IV antibiotic administration and vital observations.

The following day in HDU, the patient started experiencing a chest pain of sudden onset radiating to the upper jaws, left forearm and throbbing in nature, palpitations and started becoming diaphoretic. Blood pressure was 150/70 mmgh and pulse of 120 bpm. Emergency team was consulted, ECG readings demonstrated ST‐elevation, at lead 11, V2, and V3, (Figure 3B), serial cardiac markers, Troponin I and CK‐MB were extremely elevated, 10.0 ng/mL and 150 IU/L, respectively.

The patient was started on medication to relief acute ischemic pain these included, nitrates, morphine, antithrombotic likes aspirin and beta‐adrenergic blockade.

He was kept in HDU later with heparin 80 U/kg bolus and 8 U/kg continuous infusion and was taken for coronary angiogram which demonstrated no any coronary artery occlusion.

The patient later on started to register improvement and later discharged on medications for follow‐up.

On follow‐up, the subsequent ECG showed persistent atrial fibrillation, (Figure 3C) and patient was discharged on P2Y12 inhibitor, clopidogrel, 75 mg, and beta‐blocker, metoprolol 50 mg.

## 3 CASE DISCUSSION

There are so many possible complications of Ludwig's angina are airway obstruction, carotid arterial rupture or sheath abscess, thrombophlebitis of the internal jugular vein, mediastinitis, empyema, necrotizing fasciitis, pericardial effusion, osteomyelitis, subphrenic abscess, aspiration pneumonia, and pleural effusion.^2^

However acute MI has not been documented and the pathophysiology still not understood. By reporting a case of acute MI due to Ludwig's angina, we hope to raise the awareness in our medical community for this rare clinical entity.

A simple odontogenic problem can quickly turn fatal because of numerous critical complications, such as airway edema. As represented in this case, patients can present with exquisite pain that could yield profound sepsis if not promptly treated with antibiotics and other critical care measures, such as maintaining the airway and aggressively hydrating, spread from peritonsillar abscesses or suppurative parotitis has been documented. Ludwig's angina is commonly polymicrobial, keeping in mind the normal flora of the oral cavity. Commonly involved organisms are *Streptococcus viridians* and anaerobes like *Fusobacterium nucleatum*, *Peptostreptococcus* species, and *Actinomyces* specie.^3^

Cardiovascular complications of Ludwig's angina has not fully been documented to raise awareness to the practicing physicians. In this case report, we examine our patient who developed a cardiovascular complication due to systemic complications of Ludwig's angina.^4^

Most cases of Type 2 myocardial infarction (T2MI) are triggered by noncoronary etiologies. The response often evokes compensatory mechanisms involving both supply and demand.

T2MI can occur in patients with normal coronary arteries or in those with obstructive and nonobstructive coronary artery disease. this patient has no any prior coronary artery disease.

Distinguishing mechanisms is difficult. For example, in sepsis like in this patient, despite sufficient alterations in myocardial oxygen demand that ischemia can occur, the toxic effects of tumor necrosis factor (TNF), heat shock proteins and catecholamines, can also cause cardiac troponin, cTn release. T2MI is increasingly recognized because of the increased myocardial oxygen demand and inflammatory mediators released during sepsis.^5^

## 4 DIFFERENTIAL DIAGNOSES

The investigations confirmed the diagnosis of Ludwig's angina with MI.

In this case, it was the detailed history taking, and obvious significant bilateral submandibular swelling, accompanied by dysphonia, that raised concerns regarding the possibility of Ludwig's angina with both efficiency and certainty, ensuring the best possible outcome.

There are, however, several potential differential diagnoses to consider when assessing a patient presenting with symptoms such as those above.

These include angioneurotic edema, cellulitis, lingual carcinoma, lymphadenitis, peritonsillar abscess, salivary gland tumors, and sublingual haematoma.

Due to the rapid and potentially life‐threatening spread of this condition, early diagnosis and interventions are essential to maximize an optimum outcome for patients.

## 5 CONCLUSION

Over the past 3 decades, mortality rates for acute MI have increased significantly, one common subtype, Type 2 MI has been noticed, and defined as an MI driven by a myocardial oxygen supply and demand mismatch in the absence of coronary thrombosis.

T2MI can occur with or without obstructive coronary disease like in this patients with angiographically normal coronary arteries.

Patients with T2MI have similar or higher all‐cause mortality than patients with T1M1 in part because many studies include critically ill patients with comorbidities. They are at high risk for cardiovascular mortality and major adverse cardiovascular events.

Evidence of myocardial ischemia especially those with sepsis are likely to develop myocardial injury. T2MI is frequent and explains a significant increase in clinical practice. The mechanisms are heterogeneous, for which reason, individualized approaches to diagnosis, management, and risk stratifications are needed. A consensus is needed about how the diagnosis is established, to facilitate evidence‐based therapies geared toward improving outcomes.

### 5.1 Patient perception about this case

The patient's perception is that the swelling was something that he thought was due to tooth extraction that happened 1 month prior to admission. And he had refused to take the medications that were prescribed.

## AUTHOR CONTRIBUTIONS

**Ronald Kato:** Conceptualization; data curation; formal analysis; resources; supervision; validation; writing – original draft; writing – review and editing. **Umaru Ssebagala:** Investigation; methodology; validation; visualization. **Kainembabazi Katrina:** Project administration; software.

## FUNDING INFORMATION

There was no funding or any financial assistance that was contributed toward this manuscript.

## CONFLICT OF INTEREST STATEMENT

There is no conflict of interest amongst authors regarding the publication of this article.

## CONSENT

A written informed consent was obtained from the patient to publish this report in accordance with the Journal's patient consent policy.

## Data Availability

https://www.uptodate.com
